# Author’s Reply: Large Language Models Could Revolutionize Health Care, but Technical Hurdles May Limit Their Applications

**DOI:** 10.2196/73144

**Published:** 2025-06-25

**Authors:** Jiaming Ji, Xiangbin Meng, Xiangyu Yan

**Affiliations:** 1 Institute for Artificial Intelligence, Peking University, Beijing, China; 2 Peng Cheng Laboratory, 12 Xili Road, Shenzhen, 518055, China, 86 15896850171; 3 Institute of Disaster and Emergency Medicine, Tianjin University, Tianjin, China

**Keywords:** large language models, LLMs, digital health, medical diagnosis, treatment, multimodal data integration, technological fairness, artificial intelligence, AI, natural language processing, NLP

We thank Beltramin et al [1] for the valuable feedback and the opportunity to address the insightful comments on our Viewpoint article, “Revolutionizing Health Care: The Transformative Impact of Large Language Models in Medicine” [2]. We appreciate the their thoughtful input, which strengthens our discussion on the role of large language models (LLMs) in health care.

Our article aimed to provide a forward-looking perspective on LLMs’ potential in medicine, prioritizing conceptual insights over granular technical details. The reviewers’ points regarding multimodal data integration, image analysis, and resource allocation align with emerging research and underscore LLMs’ transformative capabilities. For example, multimodal frameworks like Med-Gemini demonstrate LLMs’ ability to process 2D and 3D medical images, extending their utility beyond conventional deep learning approaches [3].

On health care resource optimization, LLM-based methods have shown promise in enhancing operational efficiency. Techniques leveraging natural language processing can generate optimization models to improve medical resource allocation with greater accuracy [4]. Furthermore, LLMs have achieved over 90% accuracy in transforming clinical text into Fast Healthcare Interoperability Resources (FHIR) resources, facilitating streamlined data extraction and decision support [5]. While these advancements are promising, we acknowledge the need for rigorous validation and seamless integration with electronic health record systems to ensure practical adoption [6].

Regarding the second figure in our paper, our intent was to depict a generalized transformer-based framework, highlighting shared design principles across models like bidirectional encoder representations from transformers (BERT) and generative pretrained transformers (GPTs), rather than delineating their architectural differences. This schematic was meant to illustrate the broader impact of transformer-based models on medical artificial intelligence development.

Finally, our Viewpoint article does not contain factual inaccuracies, but rather provides general schematic representations of LLM architectures.

## Abbreviations

BERT: bidirectional encoder representations from transformers
FHIR: Fast Healthcare Interoperability Resources
GPT: generative pretrained transformer
LLM: large language model

## References

[1] Beltramin D, Bousquet C, Tiffet T Large language models could revolutionize health care, but technical hurdles may limit their applications (preprint). J Med Internet Res.

[2] Zhang K, Meng X, Yan X (2025). Revolutionizing health care: the transformative impact of large language models in medicine. J Med Internet Res.

[3] Yang L, Xu S, Sellergren A (2024). Advancing multimodal medical capabilities of Gemini. arXiv.

[4] Tang Z, Huang C, Zheng X (2024). ORLM: training large language models for optimization modeling. arXiv.

[5] Li Y, Wang H, Yerebakan H (2023). Enhancing health data interoperability with large language models: a FHIR study. arXiv.

[6] Ahsan H, McInerney DJ, Kim J (2024). Retrieving evidence from EHRs with LLMs: possibilities and challenges. Proc Mach Learn Res.

